# The Factor Structure of the Hospital Anxiety and Depression Scale in Orthopedic Trauma Patients

**DOI:** 10.14740/jocmr2140w

**Published:** 2015-04-08

**Authors:** Man Hung, Jerry Bounsanga, Philip Tang, Wei Chen, Christine Cheng

**Affiliations:** a Department of Orthopaedics, University of Utah, 590 Wakara Way, Salt Lake City, UT 84108, USA

**Keywords:** HADS, Depression, Anxiety, Factor analysis, Orthopedic, Trauma

## Abstract

**Background:**

Many instruments exist to assess mental disorders and anxiety, such as the hospital anxiety and depression scale (HADS). Nothing has been evaluated on the HADS factor structure for use with orthopedic trauma patients. The aim of this study was to validate the underlying structure of the HADS. Specifically, we sought to understand which of the factor structures found in the literature is appropriate for the orthopedic trauma patient population.

**Methods:**

This study included 348 patients with an average age of 49.8 years (SD: 18.4; range: 18 - 95). Confirmatory data analysis was performed to analyze the latent structure of the HADS. Akaike information criterion (AIC) was used to compare all the models, with the lowest AIC being the best fitting model.

**Results:**

We found that both the anxiety and the depression factors were highly correlated (with Pearson correlations greater than 0.700). After removing one item from each subscale, we found that a two-factor model was the best fitting one (AIC: 8,298.901); all other models had an AIC over 10,000.

**Conclusion:**

Our results support a satisfactory two-factor structure for the HADS in the orthopedic trauma patients. Further studies are needed to test for higher factor structures in larger samples and in a different population.

## Introduction

Many instruments exist to assess mental disorders and anxiety for patients. A popular instrument used is the hospital anxiety and depression scale (HADS). Zigmond and Snaith developed the HADS to assess depression and anxiety disorders among non-psychiatric patients in hospital clinics between ages 16 and 65 for both genders [1]. The HADS has two subscales: the anxiety subscale (HADS-A) and the depression subscale (HADS-D). Each subscale contains seven items for a total of 14 items in the HADS [1]. The HADS and other instruments that measure depression and anxiety can be useful when assessing patients that may be vulnerable to depression and anxiety disorders. Patients that have chronic pain and those experiencing unexpected traumatic events such as car crashes, falls, and/or accidents often report depression and anxiety. Additionally, patients experiencing chronic musculoskeletal pain are often treated for depression and anxiety [2-4]. It is crucial for clinicians that are treating patients with musculoskeletal conditions to also account for depression and anxiety [5].

Orthopedic patients are a special population as they suffer from anxiety and depression due to musculoskeletal conditions [6]. Researchers have investigated the role of the HADS in rheumatoid arthritis patients and found a high prevalence of depression and anxiety [7-10]. Additionally, depression and anxiety can negatively impact the recovery of those with musculoskeletal disorders and conditions [11, 12]. As a result, it is important to assess and study the HADS to inform physicians of possible depression and anxiety in different patient populations [13].

If the factor structure is ambiguous or unknown, clinicians may not know how to interpret the results of the assessment and may not know what intervention or treatment is appropriate for patients. For example, if we have the knowledge that the HADS instrument has two factors (e.g., depression and anxiety) and that a patient scores very high on depression, we can propose different strategies to treat this patient’s depressive symptoms. Although the HADS may be useful in determining depression and anxiety for patients, there is not a clear consensus about the factor structure of the instrument, the applicability of some of the items on the HADS for various conditions [14], and the agreement of HADS with other self-reporting instruments.

For the factor structure of the HADS, there are different opinions [14]. Depending on the medical condition being investigated, researchers have suggested one to four factors for the HADS [14, 15]. In a review of literature on the HADS, 11 of 18 studies suggested a two-factor solution [14]. Another review found that 25 of 50 studies supported a two-factor solution, five supported a one-factor solution, 17 supported a three-factor solution, and two supported a four-factor solution [15]. Others further suggested that the HADS should just be used as a one-factor general measure of distress since the study found that the symptoms of anxiety and depression were not well-separated in the HADS [16].

Research has shown that the HADS-A and HADS-D had a mean correlation of 0.56 [14]. Due to the high correlation, some scholars have suggested a single factor for calculating the total score [17, 18]. Other researchers have suggested a three-factor solution due to better fit of the data from the three factors being freely estimated [19-23]. The three-factor model includes an autonomic anxiety factor, a negative affectivity factor, and an anhedonic depression factor [20]. Researchers also raised questions about item 7 because it loaded onto two factors (negative affectivity and depression subscales). Item 7, “I can sit at ease, and feel relaxed”, an anxiety item, has demonstrated complexities in a few studies [1, 5, 14, 18]. Others have suggested the HADS should not be used in practice due to its complexities and variability of factor structure [24, 25], while some have called for additional research [26]. More recent reviews have suggested that the number of HADS factors varies widely and that the use of the HADS needs to be targeted to specific patient populations [15]. Specifically, Cosco et al [15] reported that the majority of existing research utilized factor analytic techniques from classical test theory to examine the HADS factor structure and warned that the classical test theory results would not be generalized beyond the study’s specific sample population. For results to be generalizable, item response theory methods are needed.

In reviewing the literature, we only found a single study that investigated the factor structure of the HADS for musculoskeletal patients in a rehabilitation clinic, with 55% reporting lower back pain, 20% upper or lower limb injuries, 15% cervical injuries and 10% other musculoskeletal problems. The study suggested a two-factor structure along with removing item 7, which allowed for straightforward interpretation of factors [5]. The two factors for musculoskeletal patients were interpreted as anxiety (items 1, 3, 5, 9, 11, and 13) and depression (items 2, 4, 6, 8, 10, 12, and 14). In line with the vast majority of the literature and our present study, Pallant and Bailey [5] used confirmatory factor analysis to confirm the HADS factor structure via common statistical values such as root mean square error of approximation (RMSEA) and comparative fit index (CFI). Since the patient population in our study also consisted of musculoskeletal (e.g., orthopedic) patients and our methodology was comparable to Pallant’s, we expected the factor structure for our patients to contain two factors as well. However, while Pallant et al’s patients had a wide spectrum of musculoskeletal conditions, our patients were specific to trauma. A unique study utilizing item response theory and focusing on patients with motor neuron disease [27] suggested the removal of one item from each of the two sub-scales (D8 and A11) to form two sub-scores, or the removal of three items (D8, D10 and A11) to form the 11-item high-order HADS total score. Since results from item response theory are sample independent (i.e., can be generalizable to other population), we hypothesized our results to be very close to Gibbons two-factor model.

This body of evidence from the literature suggests that the HADS factor structure is dependent on the condition and patient population [15, 28]. The sample population from previous studies primarily included adults from both genders and those having cancer, intellectual disabilities, spinal cord injury, diabetes, coronary heart disease, end-stage renal disease, schizophrenia and myotonic dystrophies. None included orthopedic trauma patients. Although all these studies essentially used factor analysis to determine the factor structure, factor analysis is a sample dependent technique from classical test theory; therefore we expected the HADS structure in the orthopedic trauma patient population to differ from what was found in studies examining populations with different medical conditions. Similar to previous studies, our population consisted of those aged at or above 18 from both genders. Despite the existence of literature on the HADS structure, there is only one study conducted in the musculoskeletal patient population and none specifically in orthopedic trauma patients. Thus, it is important to assess the HADS in the orthopedic trauma patients and to understand its internal validity. The purpose of this study was to validate the underlying structure of the HADS in orthopedic trauma patients.

## Methods

We seek to understand which of the factor structures found in the literature is appropriate for the orthopedic trauma population. The following eight models, representing the most commonly found factor structures in the literature, were examined in this study. 1) Razavi: one-factor model [17]: (items 1, 2, 3, 4, 5, 6, 7, 8, 9, 10, 11, 12, 13, and 14). 2) Zigmond: two-factor model [1]: anxiety (items 1, 3, 5, 7, 9, 11, and 13); depression (items 2, 4, 6, 8, 10, 12, and 14). 3) Moorey: two-factor model [29]: anxiety (items 1, 3, 5, 7, 9, 11, and 13); depression (items 2, 4, 6, 7, 8, 10, 12, and 14). 4) Gibbons: two-factor model [27]: anxiety (items 1, 3, 5, 7, 9, and 13); depression (items 2, 4, 6, 10, 12, and 14). 5) Friedman: three-factor model [30]: psychic anxiety (items 3, 5, 9, and 13); depression (items 2, 4, 6, 8, 10, and 12); psycho-motor agitation (items 1, 7, and 11). 6) Caci: three-factor model [23]: anxiety (items 1, 3, 5, 9, and 13); depression (items 2, 4, 6, 8, 10, 12, and 14); restlessness (items 7, 11, and 14). 7) Arving: three-factor model [31]: anxiety (items 3, 5, 9, and 13); depression (items 2, 4, 6, 8, 10, and 12); restlessness (items 1, 7, 11, and 14). 8) Dunbar: three-factor model [20]: autonomic anxiety (items 3, 9, and 13); anhedonic depression (items 2, 4, 6, 8, 10, 12, and 14); negative affectivity (items 1, 5, 7, and 11).

### Data

Data for this study were obtained from a university orthopedic center in the Rocky Mountain region of the US in 2014. Three hundred forty-eight orthopedic trauma patients were included in this study. Their mean age was 49.8 years (range: 18 - 95; standard deviation: 18.4; interquartile range: 35 - 64). There were 45.7% females, 88.8% Caucasians, and 6.61% Hispanics. Detailed sample characteristics were presented in Table 1. Data were collected on an iPad by receptionists at clinic check-ins as part of standard care. Institutional Review Board approval was obtained for the study.

**Table 1:** Sample Demographic Characteristics

	Mean (standard deviation)	Median	Interquartile range	N	%
Age	49.813	49	35 - 64	348	
Gender					
Female				159	45.69
Male				189	54.31
Race					
Caucasian				309	88.79
Black or African American				3	0.86
American Indian or Alaska Native				3	0.86
Native Hawaiian or other Pacific Islander				4	1.15
Asian				3	0.86
Other				22	6.32
Patient refused				2	0.57
Unknown				2	0.57
Ethnicity					
Hispanic/Latino				23	6.61
Not Hispanic/Latino				317	91.09
Unknown/information not available				5	1.44
Patient opts out				3	0.86

### Measures

The HADS consists of 14 items (Supplementary 1, www.jocmr.org) measuring the presence of symptoms of both anxiety and depression during the past week. Each of the two subscales, anxiety and depression, has seven items, with scores ranging from 0 to 3 per item. The maximum possible score is 21 for each subscale. A score that equals to or greater than 8 on a subscale or 11 in the whole HADS was considered abnormal [1].

### Statistical analysis

Descriptive statistics were performed to characterize the items and the patients. The SAS 9.4 software was used to check for missing data, normality, outliers, and data accuracy via plots and distributions. Data accuracy refers to whether the data values presented on our spreadsheet were correct or within the expected range.

Structural equation modeling, specifically confirmatory factor analyses with maximum likelihood estimation with robust standard errors, was performed to examine whether our data from the orthopedic trauma patient population were consistent with any one of the factor structures hypothesized by the literature. This approach was built on existing theoretical framework of the HADS and provided information about possible modifications of the relationship between the HADS items and factors. It was also robust to non-normality of data. To evaluate the psychometric fit of the HADS instrument, three indices were reported: the root mean square error of approximation (RMSEA), the standardized root mean square residual (SRMR), and the comparative fit index (CFI). Although utilizing one fit index is probably sufficient, it would be beneficial to use more than one in order to triangulate and confirm model specification. Therefore, three absolute fit indices (RMSEA, SRMR, and CFI) were reported in this study. They were selected because they are widely used in literature and are often reported in SEM studies [32, 33]. RMSEA is a non-centrality-based index, meaning it tests for how much misfit, rather than fit, is present in the model. Hence, the smaller the RMSEA value, the better the model fit. There were no strict guidelines as to what indicates adequate fit [34]. Since RMSEA ranges from 0 to 1, some suggest cutoff values of 0.01, 0.05, 0.08 and 0.1 as excellent, good, adequate and poor fit respectively [33, 35]. SRMR assesses the standardized difference between the observed correlation of model variables and the predicted correlation. Its values range from 0 to 1, with 0 indicating perfect fit and values less than 0.08 indicating good fit [36]. CFI is an incremental index, comparing the fit of the hypothesized model with the null model (i.e., the worst possible model in which all variables in the model are allowed to vary but are uncorrelated) [37, 38]. It penalizes for model complexity and its values range from 0 to 1, with 1 being the best fit, 0.95 being good fit, and 0.90 being adequate fit.

While RMSEA, SRMR and CFI are great for evaluating an individual model alone (e.g., testing whether model 1 specified above fits well, or testing whether model 2 specified above fits well), they are not appropriate for comparison of non-nested (e.g., different) models. That is, they cannot be used to compare whether model 1 is better than model 2 above, or vice versa. Hence, comparative measure of fit such as the Akaike information criterion (AIC) is needed to compare the eight hypothesized models in this study. A single AIC value alone is not meaningful; two or more AIC values are required for model comparison with lower values indicating better fit [39]. The model with the lowest AIC would be the best fitting model. Models with complicated structure (e.g., lack of parsimony) are often being penalized by AIC. All analyses were performed using MPlus 7.2 and SAS 9.4.

## Results

### HADS scores

The total HADS mean score was 10.26 (median: 8; range: 0 - 39; standard deviation: 7.71; interquartile range: 4 - 15). The mean HADS-A score was 5.26 (median: 4; range: 0 - 21; standard deviation: 4.31, interquartile range: 2 - 8). The mean HADS-D score was 5.00 (median: 4; range: 0 - 20; standard deviation: 4.08, interquartile range: 2 - 8). The Cronbach alpha reliability measures for the total HADS, the HADS-A and the HADS-D were 0.899, 0.858 and 0.822, respectively. Female had a mean HADS-A score of 5.34 and a mean HADS-D score of 5.12. Male had a mean HADS-A of 5.20 and a mean HADS-D of 4.89. There were 21.26% of patients with HADS-A of 8 or above, and 19.54% with HADS-D of 8 or above. Individual item characteristics were presented in Table 2.

**Table 2:** HADS Item Characteristics

Item code	Mean	Median	Interquartile range	Standard deviation
HADS 1	0.91	1	0 - 1	0.842
HADS 2	0.87	1	0 - 1	0.909
HADS 3	0.59	0	0 - 1	0.849
HADS 4	0.37	0	0 - 1	0.715
HADS 5	0.84	1	0 - 1	0.947
HADS 6	0.52	0	0 - 1	0.722
HADS 7	0.96	1	0 - 1	0.797
HADS 8	1.69	2	1 - 3	1.056
HADS 9	0.50	0	0 - 1	0.706
HADS 10	0.60	0	0 - 1	0.865
HADS 11	0.97	1	0 - 2	0.939
HADS 12	0.58	0	0 - 1	0.823
HADS 13	0.51	0	0 - 1	0.769
HADS 14	0.37	0	0 - 1	0.722

### Confirmatory factor analysis

All factors were highly correlated (with Pearson correlations > 0.700). Note that factor correlations are only applicable for structures having two or more factors. For the one-factor model, factor correlation is not applicable as there are no other factors for the single factor to correlate with.

Fit indices for the eight models were presented in Table 3. The two-factor and three-factor models provided adequate/good fit to the data, whereas the one-factor model provided suboptimal fit (e.g., RMSEA > 0.08 and CFI < 0.90). According to RMSEA, SRMR, and CFI, model 3 (Moorey’s two-factor model) appeared to perform the best across all indices. Nonetheless, all of the models, except model 1 (Razavi one-factor model) performed adequately.

**Table 3:** Goodness of Fit Indices for the HADS Models

Model	RMSEA	CFI	SRMR	AIC
1. Razavi: one-factor model	0.087	0.863	0.060	10,259.99
2. Zigmond: two-factor model	0.06	0.935	0.051	10,123.05
3. Moorey: two-factor model	0.046	0.963	0.038	10,071.94
4. Gibbons: two-factor model	0.062	0.943	0.050	8,298.90
5. Friedman: three-factor model	0.053	0.951	0.042	10,092.31
6. Caci: three-factor model	0.048	0.959	0.039	10,078.84
7. Arving: three-factor model	0.056	0.944	0.045	10,104.96
8. Dunbar: three-factor model	0.052	0.952	0.044	10,092.42

Since many absolute fit indices exist (besides RMSEA, SRMR and CFI), each evaluating slightly different areas of model mis-specification, it would be difficult to do pairwise comparison of all these fit indices across the models. Additionally, these absolute fit indices are not designed for comparison of different models. In order to select the best fitting model from the two-factor and three-factor models, the AIC was examined. Model 3 showed the second lowest AIC (10,071.944). Model 4 (Gibbons two-factor model) showed the lowest AIC (8,298.901) overall and hence was considered as the best fitting model; all other models had an AIC value above 10,000.

## Discussion

Our results revealed a mean total HADS score of 10.26, which is very close to the abnormal cutoff for the measure (i.e., 11). This finding is expected for patients who had recently experienced traumatic events. If we are to examine a different patient population (e.g., non-orthopedic trauma), the mean total HADS scores are likely to be lower and hence, the factor structure for HADS would probably be different. Given that the symptom level is expected to affect factor structure [15, 28], our findings for the HADS structure should be applicable to orthopedic trauma patients but not necessarily other groups.

Our study on the orthopedic trauma patient population suggested that the Gibbons’ two-factor model was the best model among all, which consists of general anxiety and depression factors. However, as suggested by Gibbons study, the standard seven-item measure of depression should be modified. Based on Gibbons work, the HADS-D subscale had a better model fitting after the removal of D8 “I feel slowed down”. Since orthopedic patients have just recently experienced a traumatic event, many may already “feel slowed down”, and this may not be a psychological disturbance but something normal as a consequence of the traumatic event. This result is also consistent with other studies on motor neuron diseases, suggesting that this item may confound the factor structure of HADS-D, at least in the orthopedic trauma patient population. Similarly, item 11 “I feel restless as if I have to be on the move” in the HADS-A subscale shows poor model fitting in Gibbons study, and indicates that a modified HADS-A subscale should be adapted to measure a patient’s anxiety status.

Considering the RMSEA, SRMR and CFI, all indicated the Moorey model to be the best fitting, it may be difficult to understand why the AIC suggested the Moorey model to be the second best while the Gibbons model is the best. Technically speaking, these two models are the same, except that the Gibbons model has excluded items 8 and 11. Since the AIC favors parsimonious model (e.g., simple model structure) and penalizes complexities (e.g., models with more parameters/items), it becomes obvious that the Gibbons model with fewer items is preferred by the AIC over the Moorey model.

Interestingly, even when all factors were highly correlated (i.e., correlations > 0.700) in the two- and three-factor models, the one-factor model was not appropriate (Table 3). These high factor correlations reflect the reality that the depression factor and the anxiety factor are related symptoms. However, they are not high enough (e.g., > 0.90) for the depression and the anxiety factors to be considered as one single factor, indistinguishable from each other.

### Limitations

Although the study found that a two-factor model was the best fitting model, this may not always be the case since the HADS is dependent on conditions and patient population. Since our study only focused on orthopedic trauma patients, there may be other patient populations experiencing trauma that needs to be examined.

### Conclusion

Gibbons two-factor model of the HADS is the most appropriate model for the orthopedic trauma population. Future studies that are considering the HADS as a screening tool should examine the best fitting model for other suitable patient populations. By assessing the HADS for orthopedic or different patient populations, both researchers and physicians have the opportunity to learn more about anxiety and depression, which may assist in effective treatment and further studies.
